# Supplementary material for: Chromothripsis during telomere crisis is independent of NHEJ, and consistent with a replicative origin
Source: Genome Res. 2019 May;29(5):737–49. doi: 10.1101/gr.240705.118 (PMC6499312; doi:10.1101/gr.240705.118)
Supplement: Supplemental Material [file supp_gr.240705.118_Supplemental_file_1.zip › contigs/annotated_contigs/DB91/contig.2.DB91_length_491_mean_cov_4.88798370672.docx]

**DB91_length_491_mean_cov_4.88798370672**

```
TCTTAATTAAATTAGCCTAATCTAATAAATGCATCATAATTAAGAGGACTGTGCCATTCTTTAGTGTTCAGCACACCAGGCATTAGATG
 >chr4:169574015-169574209 + E=4e-105
ATTTGTCCCACTTTCAGCCGTCAGTTGTCATGCTTGCTAAAAGAAAAGAAAAATGATAGCACCAGTCATCGTAGTCCCTCAAGGAATCA

TCCATCTAGTTCTTGA|TGG|ATTTTTATTGCCTTGGTTTCATATTTCACTGACTTTTAATCTGAATTTTATTTCTTTAATAAATAATT
                    >chr4:169520028-169520322 + E=2e-165
TTAAAGATGACCAAAAACATATTCTTCCTATATGTCAACATCAAGGGTCTGTAATTCCTTATTATATTGAAACTTTCTAAAAGGTCTTT

ATAAAATTCTGTTAAAATAACATATCCCAACTCAACTTCTCCAAGCCTGATCCCCAAAATAGCGAATAGCCACTCCAACTCCATGTGGC

AAGTAAGGGGTGTAACAGTGAAGAATTCAGAGTTAAGAGATGGTAGTC
```
